# Isolated Bilateral Renal Mucormycosis Masquerading as Renal Abscess in an Immunocompetent Individual: A Lesson Learnt

**DOI:** 10.1155/2014/304380

**Published:** 2014-12-18

**Authors:** Somorendro Paonam, Sananda Bag, Ravimohan S. Mavuduru, Mayank Mohan Agarwal, Arup Kumar Mandal

**Affiliations:** Department of Urology, Post Graduate Institute of Medical Education and Research, Sector 12, Chandigarh 160012, India

## Abstract

Isolated renal mucormycosis is a rare entity in immunocompetent subjects. It is usually a rapidly progressive disease with poor prognosis but it can mimic renal abscess with a protracted course.

## 1. Introduction

Mucormycosis is an opportunistic infection caused by fungi belonging to zygomycetes. Renal involvement can be a part of disseminated disease or, rarely, the sole presentation [1, 2]. They have capacity to invade vessel walls causing thrombosis and infarction [2]. In most cases the portals of entry are the nasal sinuses or the lungs [2]. It has low intrinsic pathogenicity but produces fulminant infection in immunocompromised status [3]. The mortality of different forms of mucormycosis reaches 75–100% in most series [3]. Survival for isolated renal zygomycosis is estimated to be 65% [3].

## 2. Case

A 24-year immunocompetent female with no comorbidities presented with bilateral flank pain, dysuria, pyuria, oliguria, fever for one month, and azotemia. Evaluation revealed bilateral renal and perinephric fluid collections with minimal hydronephrosis. Percutaneous pigtail was placed which drained only 5–10 mL serous fluid per day. Urine, blood cultures, and aspirates from collections were repeatedly negative for bacterial, fungal, or tubercular organisms. She remained hemodynamically stable and, however, continued to have nonrelenting fever of 38–39°C despite broad spectrum antibiotics. As pigtails drained insufficiently, bilateral open drainage of collections was performed which revealed necrotic saponified renal tissue. She developed right colonic fistula on day 2 of surgery secondary to invasion by the process for which loop ileostomy was made. On day 4, she developed necrotizing fasciitis of bilateral flank incision sites with dryness, pallor, and blackening of the wound. With high index of suspicion of angioinvasive fungal infection, parenteral amphotericin was started. Wound was debrided under anesthesia which revealed totally avascular full thickness wound with grayish moulds growing in the deep recess of wound. She died of refractory septic shock and multiorgan dysfunction syndrome. Histopathology of both the renal necrotic material and the abdominal wall tissue revealed mucormycosis (Figures 3–6).

## 3. Discussion

Isolated renal mucormycosis is a rare entity in immunocompetent subjects [4–6]. Risk factors are diabetes, diabetic ketoacidosis, and immunosuppression: neutropenia, steroids, bone marrow transplant, trauma, burns, intravenous drug use, and malnourishment [4]. Rarely healthy people are affected [4, 5]. But in northern India, healthy individuals are mostly affected which may be due to increased environmental load in this area [3]. Pathogenesis is not clear. Hematogenous angioinvasion results in vessel thrombosis and tissue necrosis [4]. The mortality of different forms of mucormycosis reaches 75–100% in most series. Survival for isolated renal zygomycosis is estimated to be 65% [4].

**Figure 1:**
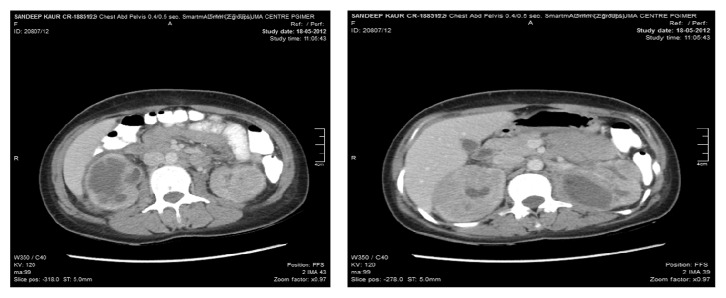
Contrast enhanced Computed Tomography scan of bilateral kidneys showing hypodense abscess like areas.

Characteristic radiological findings and early renal biopsy can confirm the disease and help in effective management of this serious disease. Ultrasonography may reveal enlarged kidneys with perinephric collections [3]. Contrast enhanced computed tomography (CECT) findings (Figure 1) include enlarged nonenhancing kidneys with absent contrast excretions, perinephric collections, and low attenuation areas suggesting intrarenal abscesses which are referred to as diffused patchy nephrograms [7].

**Figure 2:**
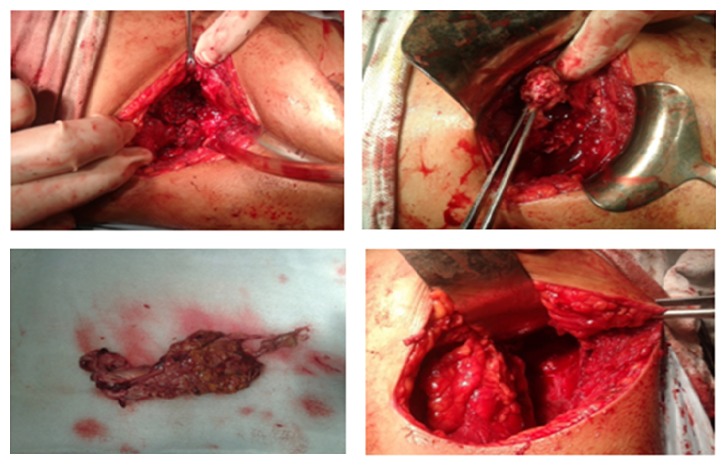
Intra operative pictures showing necrotic renal tissues.

**Figure 3:**
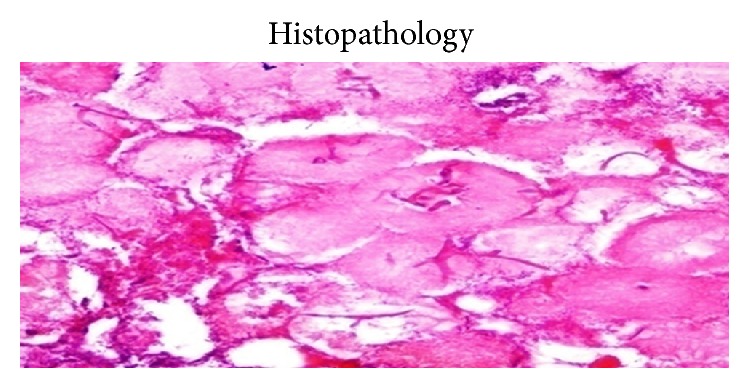
Aseptate hyphae with right angle brancing.

**Figure 4:**
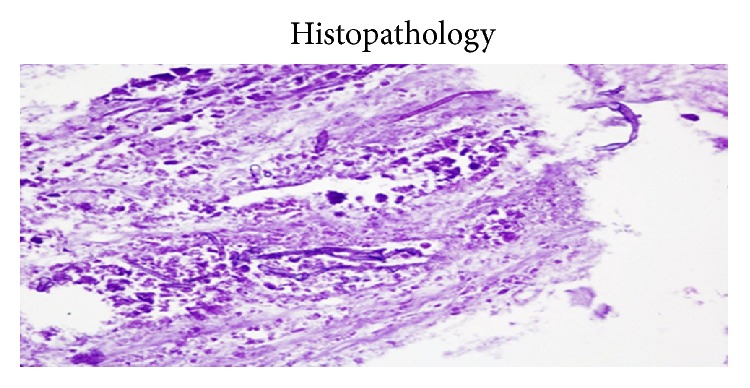
PAS staining.

**Figure 5:**
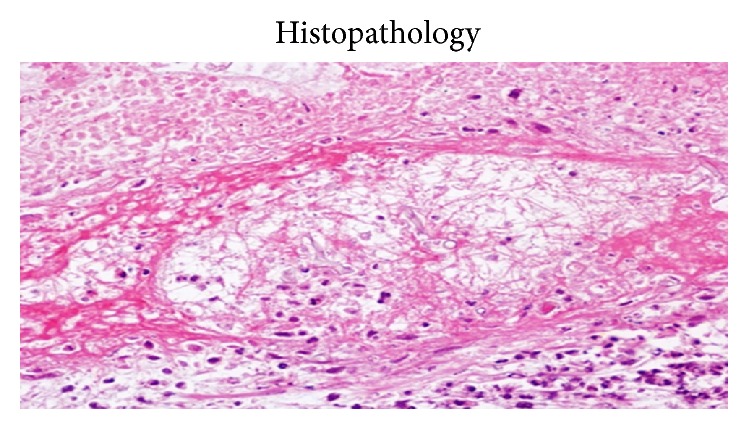
Fungal hyphae in abdominal wall tissue.

**Figure 6:**
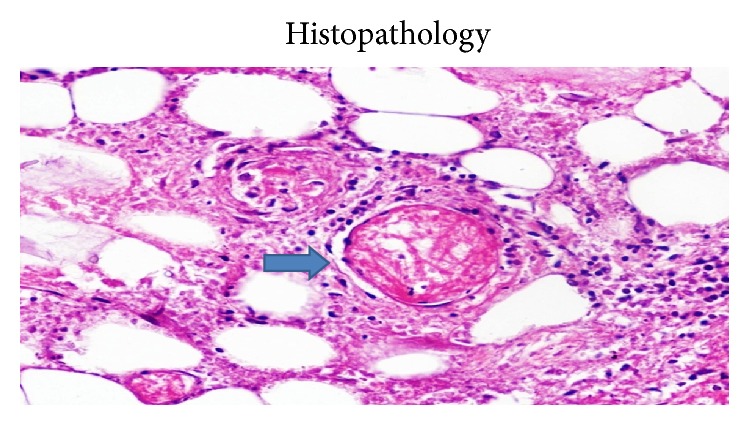
Fungal hyphae in abdominal wall blood vessel.

Clinically, most patients with isolated renal mucormycosis present with fever, flank pain, tenderness, gross hematuria, or pyuria. Blood and urine cultures are often negative and diagnosing mucormycosis almost always requires histopathologic evidence of fungal invasion of the tissues. The biopsy should demonstrate the characteristic wide (3–25 *μ*m in diameters), ribbon-like, aseptate (pauciseptate), thin walled hyphae branching at right angles. These organisms are often surrounded by extensive necrotic debris (Figure 2). Due to the thin wall they are weakly stained with Gomori methenamine silver and periodic acid-Schiff stain. Other fungi like* Aspergillus*,* Fusarium*, and* Scedosporium *may look similar to the Mucorales on biopsy. However, these molds are septate, are usually thinner, and have uniform width and dichotomous branching at acute angles. Similarly,* Candida* exists in yeasts and pseudohyphae forms with thinner hyphae, no true branching, and poor staining on routine hematoxylin and eosin but strong magenta on periodic acid-Schiff stain [4].

Successful therapy of renal mucormycosis involves a coordinated surgical and medical approach. Extensive debridement of infected and neurotic tissue, administration of amphotericin B, and reversal of underlying condition form the triad of therapy [3].

## 4. Conclusion

It is usually a rapidly progressive disease with poor prognosis. But it can mimic renal abscess with a protracted course as in our case. Diagnosis of renal mucormycosis requires high index of suspicion and identification of fungus on histopathology as clinical and radiological characteristics are nonspecific. Such subacute but relentless presentation with no microbial growth should make one suspicious of this dreadful condition. Consideration should be given to early amphotericin therapy despite nephrotoxicity.
